# Food webs for three burn severities after wildfire in the Eldorado National Forest, California

**DOI:** 10.1038/s41597-022-01220-w

**Published:** 2022-07-07

**Authors:** John P. McLaughlin, John W. Schroeder, Angela M. White, Kate Culhane, Haley E. Mirts, Gina L. Tarbill, Laura Sire, Matt Page, Elijah J. Baker, Max Moritz, Justin Brashares, Hillary S. Young, Rahel Sollmann

**Affiliations:** 1 Department of Ecology, Evolution, and Marine Biology, University of California, Santa Barbara, CA 93106-6150 USA; 2 USDA Forest Service, Pacific Southwest Research Station, Davis, CA 95618 USA; 3 Department of Wildlife, Fish, and Conservation Biology, University of California, Davis, CA 95616 USA; 4 Environmental Studies Program, University of California, Santa Barbara, CA 93106-4160 USA; 5 University of California Cooperative Extension, Bren School of Environmental Science & Management, University of California, Santa Barbara, CA 93106-5131 USA; 6 Department of Environmental Science, Policy, & Management, University of California Berkeley, 130 Mulford Hall #3114, Berkeley, CA 94720 USA; 7 Department of Ecological Dynamics, Leibniz Institute for Zoo and Wildlife Research, Alfred-Kowalke-Str. 17, 12459 Berlin, Germany

**Keywords:** Ecological networks, Fire ecology, Forest ecology, Food webs, Macroecology

## Abstract

Wildfire dynamics are changing around the world and understanding their effects on ecological communities and landscapes is urgent and important. We report detailed food webs for unburned, low-to-moderate and high severity burned habitats three years post-fire in the Eldorado National Forest, California. The cumulative cross-habitat food web contains 3,084 ontogenetic stages (nodes) or plant parts comprising 849 species (including 107 primary producers, 634 invertebrates, 94 vertebrates). There were 178,655 trophic interactions between these nodes. We provide information on taxonomy, body size, biomass density and trophic interactions under each of the three burn conditions. We detail 19 sampling methods deployed across 27 sites (nine in each burn condition) used to estimate the richness, body size, abundance and biomass density estimates in the node lists. We provide the R code and raw data to estimate summarized node densities and assign trophic links.

## Abstract (summary)

**Table:** 

Measurement(s)	Species biomass densities • Species Interactions
Technology Type(s)	Field sampling • Direct observation and rule based filters
Factor Type(s)	Forest fire burn severity
Sample Characteristic - Organism	Consumers and producers
Sample Characteristic - Environment	Mixed-conifer montane forest
Sample Characteristic - Location	Eldorado National Forest

## Background & Summary

This data set was assembled to examine how changing fire regimes affect the structure and stability of montane forest ecosystems. Historically, most dry forests of the Western United States burned frequently (return intervals <20 years) at low-to-moderate severity^1^. With Euro-American colonization, a combination of fire suppression and preferential harvest of large-diameter trees led to a predominance of young, dense and relatively homogenous forest^2,3^. The accumulation and continuity of fuels in these altered forests have led to wildfires that are both larger and much more severe than the historical norm^4^. As the planet warms, the co-occurrence of high-temperatures and low-precipitation is predicted to increase, extending the fire season and increasing the potential for megafires, not just in dry Western forests^5,6^, but globally^7^.

Megafire impacts on ecological networks in temperate and boreal forests are not well understood^8^. Megafires have a different impact on the landscape than mixed-severity fires. Mixed-severity fires create complex landscapes of heterogeneous habitats that span large gradients in patch size^9^. Megafires, characterized by size (areal extent >10,000 ha) and severe burn intensities, create homogenous landscapes causing major shifts in community structure and composition (not to mention economic loss and health risk for humans)^8^. For instance, megafires often alter microclimates, impacting tree reestablishment and favoring invasion by exotic species^8^. These alterations can cause long-term changes in vegetation type^10^, including in adjacent unburned forests; ultimately impacting the abundance and distribution of wildlife^11,12^. We use a recent megafire in the Sierra Nevada range to explore the response of ecological communities to variation in burn severity.

The King Fire was a 2014 megafire in the Eldorado National Forest, California. Located 200 km Northeast of San Francisco, the Eldorado National Forest is 320,000 ha spanning 3,000 m in elevation and contains chaparral, conifer, fir and subalpine habitats which receive about 142 cm average annual precipitation. Ignited by arson, the King Fire burned from 13-Sep to 10-Oct 2014 and consumed 39,545 ha (an area twice the size of Washington, D.C.), most of that during the first five days. Drought conditions led to high fuel consumption and intense fire behavior^13^. Half of the King fire burned at high severity, including a contiguous 13,683 ha patch^14^. Its large size, high severity, and wildland location (rather than a more developed area) make the King Fire an ideal setting to examine the impacts of fire severity on ecological communities in lower montane western coniferous forests.

Three years post-fire, we used intensive field sampling to assemble food webs in forest patches that experienced different burn severities. Food webs trace the flow of energy through ecosystems, illustrating structures and concepts such as indirect interactions, trophic cascades and apparent competition. To construct these trophic networks, we provide high resolution data on identity, abundance, biomass and feeding linkages for all organisms surveyed in each of the three burn severities sampled. No food web is perfect^15,16^, but the food webs presented here were assembled with the intent of improving on some shortcomings in terrestrial webs including lumping invertebrates^15,17^, omitting vertebrates^18,19^ and small size^20,21^. To our knowledge, the data provided describe the first comprehensive community-scale food webs replicated across burn categories.

**Fig. 1:**
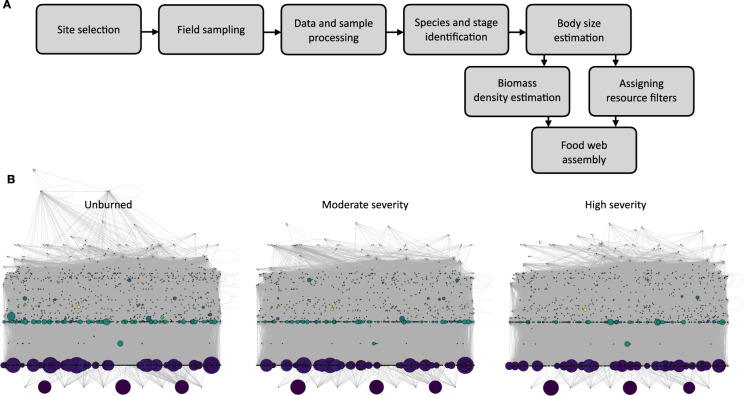
Assembly workflow and food webs for three burn severities in the Eldorado National Forest, California. (**A**) Workflow for assembling quantitative food webs detailed in the manuscript. (**B**) Food webs, including biomass for three burn severities in the Eldorado National Forest. From left to right webs depict trophic networks for unburned sites, low-to-moderate severity burn sites and high severity burn sites. Circles (nodes) represent species life stages or plant parts. Node size is log biomass density. Inferred nodes that were not observed received a dummy biomass of 2 for visualization (added to all nodes to keep relative sizes consistent). Nodes are colored by rough groups (e.g. conifers, coleoptera, non-living, etc.). Vertical height is short-weighted trophic level. X-axis position is random. Node color and X-axis position are conserved across webs. Node trophic level varies across webs.

We are using the data to explore community responses to fire severity across trophic levels. We recently explored changes in spatial use by bats as a function of vegetation structure and availability of nocturnal flying insects^22^. Bats appeared resilient in response to fire. While vegetation and prey availability (e.g. moth biomass and richness) varied greatly, most bat species showed no response to burn severity^22^, likely due to their highly mobile nature. We also examined the response of small mammal communities to fire-driven changes in vegetation structure. Small mammal abundance was constant across burn severities, despite decreasing richness. Remarkably, these changes in small mammal community structure were predictable from interactions between vegetation structure and small mammal foraging and nesting habits. Finally, our preliminary analyses of food webs for each burn category (Fig. 1) suggest that network stability significantly decreases with burn severity. Even before correcting for stability gains from decreases in size, severely burned food webs are twice as unstable as moderately burned food webs. These data should be valuable to both network topologists and community ecologists.

## Methods

### Site selection

**Fig. 2:**
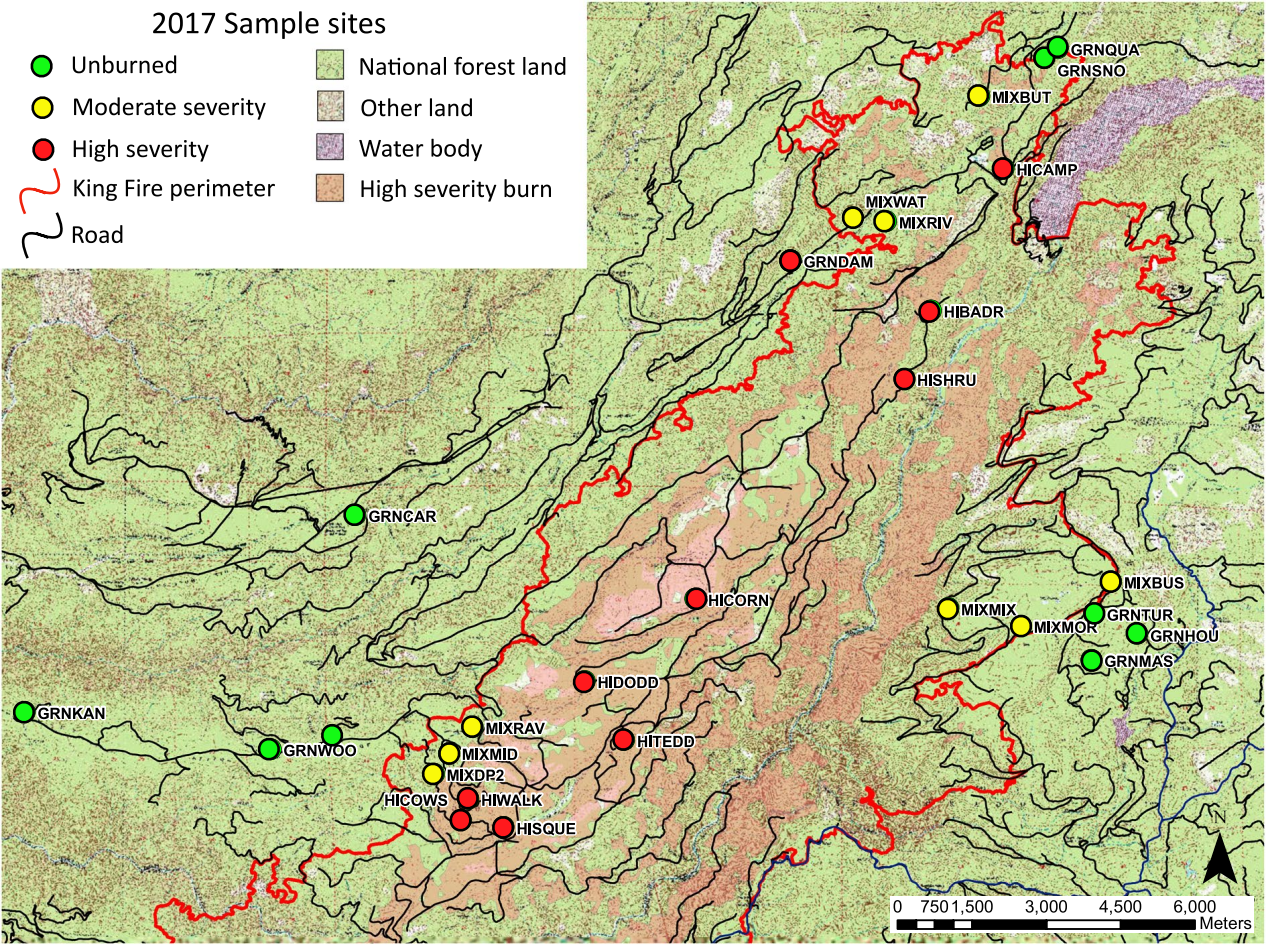
Sampling sites map and King Fire perimeter. We sampled 27 sites total in the Eldorado National Forest, California, three years after the King Fire, nine in each burn category: Unburned, moderate severity, and high severity. Features not indicated in legend are typical of topological maps.

Our study focused on the mixed-conifer zone of the Sierra Nevada which is dominated by Ponderosa pine (*Pinus ponderosa*), Jeffrey pine (*Pinus jeffreyi*), Incense cedar (*Calocedrus decurrens*), White fir (*Abies concolor*) and California black oak (*Quercus kelloggii*). Common shrubs include Deer brush (*Caenothus integerrimus*), Mountain whitethorn (*Caenothus cordulatus*), Greenleaf manzanita (*Arctostaphylos patula*) and Prostrate ceanothus (*Caenothus prostrates*). Study sites in the Eldorado National Forest near Placerville, CA (38°45′N 120°20′W), were in and near the area burned during the King Fire (Fig. 2).

We sampled sites in the mixed-conifer zone between 4000–6000 ft in three burn categories: unburned, low-to-moderate severity and high severity. We selected sites that occurred in similar pre-burn habitat (moderate to dense conifer forest) based on remotely-sensed vegetation class data from the California Wildlife Habitat Relationships program (CWHR)^16^. No site experienced wildfire or controlled burns in the preceding century^23^. Burn categories were based on remote sensing relative differenced Normalized Burn Ratio (RdNBR) canopy cover calibrated for the Sierra Nevada^24^. We validated these burn categories as meaningful and discrete with remote sensing (immediately post fire) and field data (3 years post-fire). Monitoring Trends in Burn Severity (MTBS) maps classify burn severity with Landsat reflectance imagery of pre-fire and post-fire conditions at 30-m resolution^25^. MTBS assigns pixels a value based on burn severity: 0 = outside the burn boundary; 1 = unburned-low severity within the fire perimeter; 2 = low severity; 3 = moderate severity; 4 = high severity). At each site, we determined the mean MTBS value of pixels in the small-mammal trapping grid (90 × 90 m) and a 225 m buffer (the largest home range diameter for small mammals we captured)^26^ extended on all sides. Immediately after the fire, the three burn categories differed significantly in tree cover and remote sensing scores of burn severity^25^ and are still different in tree surveys three years later.

We sampled 27 sites, each 4 ha in size. To minimize site-specific influences, we paired sites across treatments to account for elevation, slope, pre-burn vegetation characteristics, pre-burn forest management, ownership, and soil type. Each burn category received nine sites. We blocked sites across burn severities (one site in each burn category per block). Site locations were chosen for accessibility, but all sites were at least 50 m from access roads and at least 200 m apart (sites were >1 km apart on average). We also excluded areas that experienced or were scheduled to experience salvage logging post fire. Occasionally, field conditions at a site location did not align with remotely sensed data classifications, a site was not large enough for homogeneous sampling plots or was dominated by slopes >30 degrees. In these cases, sites were moved to nearby locations that satisfied the site selection criteria.

#### Sampling design

**Fig. 3:**
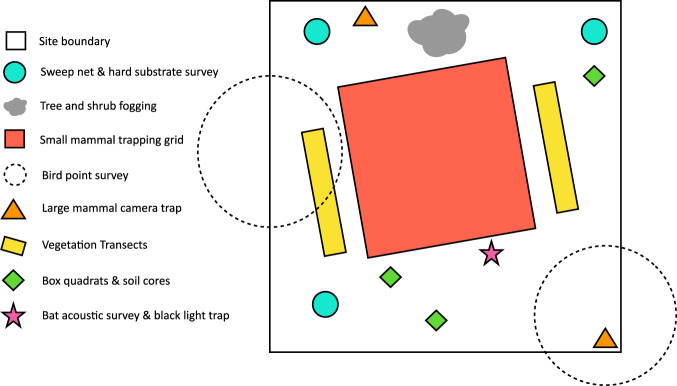
Representative map of sampling design. We employed 19 different methods to estimate the richness and biomass density of organisms in the Eldorado National Forest, California, three years after the King Fire. Methods were conducted entirely within or centered within the 200 × 200 m site perimeter. Methods were paired in space and time when useful (e.g. black lights and acoustic bat surveys), and separated when necessary to avoid interference (e.g. small mammal trapping grid and vegetation transects). All methods at a site were conducted over 4–5 consecutive days.

In this study we report the body size, abundance and biomass density of plants and free-living animals using a variety of methods in three different fire severities. Each site consisted of a 200 m × 200 m plot (4 ha) around which sampling methods were organized (Fig. 3). We collected data for organisms with 19 different sampling methods that were scaled to the abundance and body size of targeted organisms. Some methods were not performed at all sites, and some methods were pooled across sites (within treatments) due to low sampling success. Below, we explain each sampling method and detail any variation in its application across sites. To minimize seasonal effects, we sampled all sites in a block at the same time, over 4–5 continuous days, between late June and early September 2017. Weather was consistently hot and dry during this period with no pronounced variation. All animal survey procedures were approved by UC Davis IACUC (protocol number CA-17-451736) and carried out under CDFW permit #SC-3638.

#### Species inclusion

To evaluate the effects of wildfire-burn severity on community structure we assembled a list of organisms and life stages encountered during our sampling of the Eldorado National Forest. Every animal in our list was broken into ontogenetic life stages. Plants were broken into constituent parts (e.g. leaves, roots, seeds) rather than stages. Every organism in our lists had at least one life stage observed by this study in the Eldorado National Forest. Not all life stages in the list were directly observed, many were inferred. These life stages were suspected to occur in the habitat but were not observed (or quantified) because (1) it was impractical (e.g. mycorrhizae), (2) our sampling methods did not capture them (e.g. larval insects in plant tissues) or (3) they could not be identified to species (e.g. arthropod eggs). Stages (not species) were excluded when they did not occur in the terrestrial habitat (e.g. aquatic larvae). Stages (or parts) were lumped when they could not be distinguished in terms of their resources or consumers (e.g. parasitoid wasp eggs, larvae and pupae). Unobserved stages were omitted from a burn category community if they were feeding (e.g. not eggs) but did not have any resources. All life stages, observed or not, received a body size estimate.

This approach has several benefits. First, it fills life-cycle gaps without artificially inflating species richness (e.g. having a node labelled “bird eggs”). Second, unobserved life stages may not be major biomass contributors, but they do make important contributions to food-web structure and population dynamics. Including life stages without abundance information is useful because it allows their inclusion in analyses of network structure, informs consumer-resource body-size ratios and allows comparisons to other food datasets organized around body size, but lacking abundance estimates.

A comprehensive food web required the inclusion of non-living nodes like detritus which is an important resource for many consumers. Fire creates strong spatial structure in woody detritus, so we collected mass-density information on it and partitioned detritus into types (e.g., woody vs. leaf) and size classes. We did not collect mass-density information on carcasses but treated them similarly by partitioning them into logarithmic size classes to reflect their availability to different consumers (e.g., tick carcasses vs. deer carcasses). We have also included the biotic products honeydew and dung due to their importance to certain consumer groups in the system. We have not done so here but future efforts may wish to include nutrients as resources for plants to capture gradients and competition. Finally, analyses may wish to augment our lists by explicitly including fire as a consumer/herbivore as opposed to an external force shaping treatment effects.

Below we describe the methods used to quantify the richness, abundance, and biomass density of these organisms in each of our burn categories. Unless noted, sampling effort did not vary with burn category.

#### Species resolution

Most entries in the species list represent ontogenetic stages or constituent parts. With the exception of a few nodes (i.e. algae, moss, lichen, mycorrhizae, saprophytic bacteria, saprophytic fungi and nematodes) this ecological resolution is consistent throughout the list. Taxonomic information was assigned using the Global Biodiversity Information Facility database^27^, but taxonomic resolution varies. Vertebrates are identified at the species level. Invertebrates, while distinguished as morphospecies, were not identified below the family level in many cases. Despite not always being identified to the lowest taxonomic category, entries represent life stages of distinct species or groups, and are accompanied by all the taxonomic information we could provide at that level of resolution.

#### Biomass density estimation

Each species observed in our sampling methods received a biomass density estimate. Biomass density was estimated as the product of mean individual body mass and density. We estimated body-mass either by weighing individuals directly, or conservatively estimating their mass volumetrically. For the latter, we measured the length (tip of abdomen to tip of head), width (max width of body excluding appendages) and depth (max depth of body excluding appendages) of individual organisms and converted into an approximate volumetric shape (e.g. ellipsoid, cylinder, hemisphere, etc.)^28,29^. Mass was estimated by multiplying this volume by a tissue density of 1.1 g/mL^30–32^. Body sizes for stages that were not directly observed were inferred from published records and databases. Density estimates were derived from the sampling methods discussed below and varied with burn category. Mean and standard errors were derived from the nine replicate sites within each treatment, unless otherwise stated. Due to the brief sampling window at each site, these estimates should be regarded as point estimates rather than integrated over time.

### Plant sampling

#### Vegetation transects

To estimate density and biomass of trees, shrubs, and ground cover, we conducted vegetation transects at each site. Transects were located within each 200 m × 200 m plot but were offset from other sampling methods by 10 m to avoid trampling. Transect direction was chosen at random and two transects were run in parallel. Each transect was 50 m in length but width (and distance between transects and sampling area) varied with sampling method as detailed below.

#### Ground cover

To estimate plant ground cover, we surveyed 1 m^2^ quadrats at 5 m intervals on each 50 m transect. Summed, this gave a pooled ground cover sample area of 20 m^2^ at each site. Within each quadrat we estimated the percent cover and average height of grasses, forbs, woody litter, soft-loose and soft-rooted litter, shrubs, and trees. We identified small trees, shrubs and dominant forbs (i.e., forbs with the greatest cover at a site) to species. We then used cover area and height to estimate a volume for each species or group. Volumes were later multiplied by taxon-specific measurements of mass-to-volume ratios derived from vegetation box quadrats to obtain biomass estimates for each ground cover species or group.

#### Canopy cover

To estimate canopy cover, above each of the 20 ground cover sampling locations, we identified each tree species overhead and estimated its absolute canopy cover. This gave a pooled canopy cover sample area of 20 m^2^ at each site. Canopy cover estimates were incorporated into arthropod biomass density estimates from fogging.

#### Trees

To estimate tree density, we identified and measured all trees that exceeded 15 cm diameter-at-breast-height (DBH) within a 15 m wide band extending the length of each 50 m vegetation transect. Small trees (DBH <15 cm) were assessed with shrubs (see below). This gave us a total sample area of 1500 m^2^ for measuring tree density and DBH at each site. We estimated tree mortality at each site by binning trees encountered on transects into three health categories: healthy, unhealthy and dead. These categories apply to individual trees and are independent of the remote sensing categories for burn severity used for mapping sites. Health categories for individual trees were as follows: Healthy (at least 75% of needles intact), Unhealthy (between 25% and 75% of needles were either missing, yellowed or burned), and Dead (more than 75% of needles were missing, yellowed or burned). Dead trees were incorporated into the species list as large woody detritus. We estimated individual tree biomass by combining DBH-to-biomass estimates for total biomass as well as proportional biomass estimates for various plant tissues. This allowed us to create species-specific biomass estimates for individual trees that also integrated biomass loss from damage or death.

To estimate above-ground biomass of individual trees we identified them and applied the DBH-to-biomass conversion from Jenkins *et al*.^33^1:

*β*_0_ and *β*_1_ are species-specific parameters from Jenkins *et al*.^33^. This allowed us to estimate the proportion of the tree remaining. When then estimated the ratio of various tissue type biomasses (foliage, coarse roots, stem bark, stem wood) to total above ground biomass:

2 $$ratio=Exp\left({\beta }_{0}+\frac{{\beta }_{1}}{DBH}\right)$$

*β*_0_ and *β*_1_ are tissue-specific parameters that also differ for hardwoods and softwoods^33^. Next, we estimated the probable full-height of damaged trees from their DBH:

3 $$Full\;height=Exp({\beta }_{0}+({\beta }_{1}\ast ln(DBH))+({\beta }_{2}\ast ln{(DBH)}^{2})+{\beta }_{3}\ast ln{(DBH)}^{3})$$

*β*_0_, *β*_1_, *β*_2_ and *β*_3_ are species-specific parameters^33^. We were unable to identify 18 individual trees, for all parameters, these were assumed to be members of Pinaceae, which comprised 95% of identified individuals. With these estimates we binned individuals into four categories and estimated their biomasses: Intact alive, intact dead, damaged alive, damaged dead. To estimate the total biomass for intact alive trees we added estimated root biomass to the estimated total above ground biomass. To estimate the total biomass for intact dead trees we removed estimated foliage biomass from total above ground biomass and added estimated root biomass. To estimate the biomass for damaged but alive trees, we corrected the biomass estimate for each tissue type by the proportion of tree remaining. The biomass of damaged alive trees was estimated as the sum of the corrected biomass estimates for these tissue types. To estimate the biomass of damaged dead trees, we corrected the biomass estimate for each tissue type by the proportion of tree remaining. The biomass of damaged dead trees was estimated as the sum of the corrected biomass estimates minus foliage biomass.

##### Understory shrubs and small trees

To estimate the density of shrubs and small trees, we counted all individuals within a 5 m band extending along the vegetation transect. This gave us a sample area of 500 m^2^ for understory shrubs and small trees at each site. Shrubs were included if their main stem occurred within the 5 m band of the transect, and it exceeded 50 cm in at least one dimension (length, width, or height). Small trees were included if their bole occurred within the 5 m band transect and DBH >1 cm. We estimated shrub volume (length × width × height) and biomass density by combining volume and density estimates with taxon-specific measurements of mass-to-volume ratios derived from vegetation box quadrats. We estimated above ground biomass for small trees using species-specific DBH-to-biomass conversions^33^.

##### Understory plants

To estimate the volume and cover of understory vegetation we used three-dimensional box quadrats. These box quadrats consisted of a canvas-walled rectangular box with the floor panel removed, that was placed over the understory to be surveyed. The canvas walls prevent arthropods from escaping as they were also used in arthropod sampling. Each box quadrat measured 1 m × 0.5 m × 0.5 m, covering a ground area of 0.25 m^2^. At each site, we placed box quadrats at three locations representative of the dominant vegetation. This gave a pooled sample area of 0.75 m^2^ at each site. To generate a volume estimate, within each box we estimated the percent cover and maximum height of each plant species. Leaf litter was treated separately but also quantified. Then, using a Velcroed flap as access (designed to prevent bugs from escaping), we collected and weighed all the above ground plant biomass, separated by species and type (litter, branches, leaves, etc.). A subsample of material (up to 1 kg) from each plant species and type was retained and returned to the station to extract the associated arthropods via Berlese funnels (detailed below).

For each plant species measured in the vegetation box samples, we estimated a mass-to-volume ratio. We estimated the biomass of understory plants by combining our mass-to-volume ratios with volume estimates from ground cover transects. For taxa present in ground cover transects but not vegetation boxes, or with fewer than three measurements of mass-to-volume ratio, we pooled measurements from higher taxonomic levels. For example, if there was only one measurement of species A, but two measurements for a congener, species B, we used measurements for both species *A* and *B* to represent the mass-to-volume ratio of each. In this way, we estimated mean mass-to-volume ratios for every species-type encountered in the shrub and cover transects.

#### Coarse woody debris

To estimate the mass density of woody detritus we quantified it according to the method detailed in Waddell (2002). Specifically, we used the center of the vegetation transect as a line-intercept for woody detritus. Woody debris was recorded if its longitudinal axis intersected the transect line, its diameter at the point of intersection was ≧ 12.5 cm; it was ≧ 1 m long and it was not decayed to the point of disintegration. For each piece of woody detritus, we measured the diameter at both ends, the length, and the stage of decay. Length was measured only for the portion exceeding 12.5 cm in diameter. We estimated the volume (m^3^) for each piece of debris:

4 $$V{m}^{3}=\left.\frac{(\pi /8)({D}_{s}^{2}+{D}_{L}^{2})l}{10,000}\right)$$

${D}_{S}^{2}$ is small diameter (cm), ${D}_{L}^{2}$ is the large diameter (cm) and *l* is the length (m)^34^. We converted this estimate to a volume-density (m^3^ ha^−1^) estimate:

5 $${m}^{3}h{a}^{-1}=\left(\frac{\pi }{2\;{L}_{t}}\right)\left(\frac{V{m}^{3}}{{l}_{i}}\right)f$$

*L*_*t*_ is the combined transect length (100 m), *l*_*t*_ is the length of the individual piece, *f* is a conversion for area (10,000 m^3^ ha^−1^)^34^. Finally, we converted this is a dry-weight biomass density (kg ha^−1^):

6 $$kg\;h{a}^{-1}=({m}^{3}h{a}^{-1})\left(\frac{1000\,kg}{{m}^{3}}\right)SpG\ast D$$

*SpG* is a specific gravity estimate and *D* is the correction for the state of decay^34^. Specific gravity can be a species-specific estimate. Woody debris is difficult to identify to species so we applied a single specific gravity estimate to all debris weighted by the relative abundance of tree species in our surveys. We used a weighted specific gravity of 0.382, estimated by multiplying the mean specific gravity of Pinaceae (0.372) by their relative abundance (0.947) and adding that to the specific gravity of Quercus (0.566) multiplied by their relative abundance (0.053). We applied the weighted means to the decay corrections in Waddell (2002).

#### Species of uncertain identification

Some plant identifications were difficult, particularly at burned sites, where some individuals were identified to genus or family. To assign species identities to individuals classified a higher taxonomic rank at a focal site, we first randomly selected a proxy site from the nine unburned sites. This proxy approach is possible because the composition of burned and unburned sites was similar before the fire. Proxy sites were randomly selected from among all the unburned sites because the spatial grouping didn’t lend itself to maintaining distinctions between blocks. Using the species abundance distribution of the focal plant taxon at the proxy site, we randomly assigned (with replacement) plant species identities to unidentified individual plants at the focal site. We repeated this random sampling 999 times, then estimated the resulting mean species abundance and bootstrapped standard errors at the treatment level.

### Invertebrate sampling

Given the diversity of invertebrates in the ecosystem, and the biases associated with each invertebrate sampling method, we employed several methods to quantitatively survey the entire community.

#### Arthropods on understory plants

To estimate the density of arthropods on understory plants we collected them along with plant material in the box quadrats. Up to 1 kg of plant material of each species was bagged inside box quadrats and transported to the field station for processing. Insects were extracted from the plant material in Berlese funnels that were hung in the shade at ambient temperatures and powered with 60 W frosted-incandescent bulbs. All plant samples were processed in funnels for 72 hours, after which they were checked twice a day (collecting jars changed). After no additional arthropods had been collected for 24 hours samples were removed. Arthropods were placed in ethanol for later identification. After identification, arthropod densities were estimated as the product of the arthropod-to-volume ratio for each plant and the total volume of each plant estimated from transects. We collected 4543 individual arthropods from 170 morphospecies associated with plant material from box quadrats, processed in Berlese funnels.

#### Soil-dwelling arthropods

To estimate the density of soil-dwelling arthropods we collected soil cores. Alongside each box, we collected four cores, each 10 cm in diameter. Two shallow cores were sunk to a depth of 5 cm, giving a combined (6 replicates total) sample area of 0.0471 m^2^ at each site. Shallow cores, which targeted small arthropods (e.g. collembola, diplura, acari), were collected and transported back to the field station for processing in Berlese funnels. Soil samples were processed in Berlese funnels in the same manner as plant material. Two deep cores were sunk to a depth of 15 cm, giving a combined (6 replicates total) sample area of 0.0471 m^2^ at each site. Deep cores targeted large invertebrates (e.g. lumbricidae, myriapoda) and were processed by hand in the field. Any large invertebrates encountered were placed in ethanol for later identification. After identification, densities were estimated as the quotient of counts and area sampled. We collected 690 individual arthropods from 57 morphospecies from soil cores.

#### Arthropods on hard substrate

To estimate the density of arthropods like wasps and spiders on tree trunks, rock and logs we visually surveyed these hard substrates. In three locations at each site, we surveyed all hard substrates within a cylinder 5 m in diameter and 2 m in height. The total sample area at each site was 58.9 m^2^. All invertebrates larger than 2 cm in length were collected and fixed in ethanol for later identification. After identification, hard substrate densities were estimated as the quotient of counts and sample area. Hard substrate surveys yielded 616 individual arthropods from 74 morphospecies.

#### Understory arthropods

To estimate the arthropod densities associated with understory shrubs at each site we supplemented box quadrats with sweep net surveys. We used nets with a 38 cm diameter opening (Bioquip) to sweep the same 5 m diameter area as the hard substrate surveys. Three replicates at each site gave a total sample area of 58.9 m^2^. Net sweeps were performed at a constant pace of 2 arcs per meter. All invertebrates collected were fixed in ethanol for later identification. After identification, arthropod densities were estimated as the quotient of counts and sample area. Sweep net surveys yielded 706 individual arthropods from 168 morphospecies.

#### Canopy arthropods

To survey arboreal arthropods, we used a thermal fogger to loft insecticidal fog into the tree canopy. While this method can sample a large area, we were hampered by permit and accessibility issues at some sites. As a result, we sampled a representative subset of 12 sites (6 unburned, 3 high severity, and 3 low-to-moderate severity) with canopy fogging. Chemical fogging remains the most widely used method for sampling canopy arthropods^35–37^. It is notoriously difficult to assess canopy arthropods with any other method^35^. We used an IGEBA TF-35 Thermal Fogger to disperse a pyrethrum-based insecticide (EverGreen Crop Protection EC 60-6) into the forest canopy. During each fog we dispersed 4 L of solution (a 7% concentration of insecticide in a water-based carrier-dispersant) in 10 minutes over two representative trees and one live shrub at each site. Live trees were sampled at unburned sites, dead trees were sampled at high severity sites, and one dead and one live tree were sampled at low-to-moderate severity sites. Fogging was done under low-wind conditions allowing the thermal fogger and temperature gradients to lift the fog into the canopy. White 2.25 m^2^ tarps were placed beneath the fogged area to collect insects. The number of tarps used varied with the size and shape of the canopy. After an optimal elapsed drop-time of 120 minutes^37^, arthropods were collected from tarps and placed in ethanol for later identification. To estimate site-level arthropod abundance using canopy fogging, we estimated the density per square meter of tarp for each morphospecies and habitat type (living tree, dead tree, or shrub), then multiplied these densities by the total area covered by each habitat type. The area covered by each fogged habitat type was obtained from canopy cover transects (trees) and shrub transects (shrubs). Mean and standard errors for density estimates were estimated using the 3 (or 6) sites per treatment. Canopy fogging yielded 16,353 individual arthropods from 489 morphospecies.

#### Strong-flying insects

To estimate the diversity and relative abundance of strong-flying arthropods, we utilized blacklight traps (Miniature Downdraft Blacklight (UV) Trap Model 912). We supplemented our sampling with blacklight traps, because strong flying arthropods (e.g. vespid wasps) may not be adequately sampled by fogging or sweep netting. Black lights were deployed at sites prior to dusk on the third night of bat acoustic surveys (see below). Each site received one trap and black lights were not deployed on rainy or windy nights. Samples were collected the following morning. To preserve their integrity for later identification, lepidoptera samples were removed and frozen, other flying insects were fixed in ethanol. Black light traps collected 5,508 individuals from 279 morphospecies.

Black light samples were only used to estimate densities for those species not regularly captured with other methods. Two hundred fifteen morphospecies were only found in black light samples. Black light traps, which can sample large areas, are an efficient means of generating diversity estimates for nocturnal flying insects. However, black light traps generate activity densities rather than absolute densities, over sample areas that vary within and between nights. To convert counts to density estimates for morphospecies collected only in black lights we paired them with an analogous species (based on taxonomy and body size). These density analogues were captured at the same site in both black lights and at least one other method with explicit absolute densities. We estimated the mean relative abundance ratios of these species in black lights across all sites in which they co-occurred. We then multiplied the density of the ecological analog by these abundance ratios to estimate densities of all species that were collected only in the black light traps.

#### Larval biomass density

To estimate the density of larval Formicidae, lepidoptera and coleoptera, we partitioned them according to the relative abundance of their adults present in the same treatments. Larvae for many hemimetabolous invertebrates can be difficult to identify without molecular methods. When larvae could not be identified to species, they were binned into categories of Formicidae, lepidoptera and coleoptera. We then collected body size information and density estimates for these stages. Larval densities were then partitioned according to the relative abundance of adults within each treatment. Adults were eligible to receive larvae if (1) they belonged to the same taxonomic rank as the larvae, (2) they did not already have any observed larval stages, (3) there were available resources for their larvae in the treatment.

### Vertebrate sampling

#### Small mammals

To estimate the density of mammals less than 2.5 kg we used live traps uniformly distributed over a 90 m × 90 m grid^38^. We placed 100 traps 10 m apart, alternating between large (7.5 cm × 9 cm × 23 cm) and extra-large Sherman traps (10 cm × 11.5 cm × 38 cm)^39^. Traps were baited (a mix of oats, peanut butter, bird seed and molasses) and covered with natural materials for insulation. Traps were locked open and pre-baited for three days and then operated for three consecutive nights. Traps were closed during the day due to high daytime temperatures and lack of shade, particularly in the high severity burn areas. Traps were re-opened in the late afternoon. Captured mammals were identified to species (or individuals during recaptures), weighed, measured and fit with ear tags for future identification, then released. In 7,906 trap nights (adjusted for trap failures), we had 988 captures of 11 species of small mammals.

Live-trapping and marking of small mammals allowed us to estimate their abundance using spatial capture-recapture (SCR) models^40^. These models use individual detections in combination with detection locations (here, location of a given live trap within the grid) to estimate density while accounting for imperfect detection and variation in detection due to variability in individual exposure to the trapping grid. When data are collected across several trapping grids, as in the present study, the joint modeling of all data allows estimation of grid-level covariate effects on density^41^. We used this framework to analyze data from all 27 plots jointly and allowed for density to vary according to burn category of a site (unburned, low-to-moderate severity or high severity), so that, for example, all unburned sites had the same density of a given species. If a species was never caught in a burn category, we fixed its density (and consequently, its biomass) to 0 for all sites in that burn category.

Because trapping data for most species was too sparse to fit species-specific models, we grouped ecologically similar species and built models that shared parameters among grouped species. In this manner, we analyzed the joint data of mice and chipmunks: Brush mouse (*Peromyscus boylii)*, Deer mouse (*Peromyscus maniculatus*), Pinyon mouse (*Peromyscus truei*) and Western harvest mouse (*Reithrodontomys megalotis*), Yellow-pine chipmunks (*Tamias amoenus*), Long-eared chipmunks (*Tamias quadrimaculatus*) and Shadow chipmunks (*Tamias senex*).

In the mouse model, Brush mouse and Pinyon mouse densities were assumed to have the same response to burn category (but not the same densities), whereas Deer mice were allowed a different response to burn category. This choice was made based on capture frequencies of species in the different burn categories. Pinyon mice and Western harvest mice had a fixed density of 0 in low-to-moderate and high severity burn sites. All species shared the same detection parameters.

Even combined, the chipmunk data captures were too sparse to estimate species specific densities. We therefore treated all chipmunks like a single species to estimate overall chipmunk density, then calculated species specific densities by multiplying overall density with the proportion of individuals of a given species in the data. For example, if species A made up 50% of all individuals in the data, we would calculate density for species A by multiplying overall chipmunk density by 0.5. This model entails the assumption that the effect of burn category on density was the same for all chipmunks, which seems reasonable based on capture frequencies.

We built species-specific SCR models for California ground squirrels (*Otospermophilus beecheyi*) and Dusky-footed woodrats (*Neotoma fuscipes*); for the latter we fixed density to 0 in high severity sites. Northern flying squirrels were only caught 3 times at unburned sites, so we set its density to 0 in low-to-moderate and high severity sites, and used a published density estimate from a Sierra Nevada site^42^ for unburned sites. Because individuals of Trowbridge’s shrew (*Sorex trowbridgii*) were never recaptured and had high rates of trap mortality, we estimated their abundance using non-spatial removal models^43^ for unburned and moderately burned sites and set their abundance to 0 at high severity sites. We transformed abundance to density by dividing it by the 8100 m^2^ area (90 × 90 m) of the live trapping grid.

We fit SCR models in R using the package “secr” ver. 3.1.3^44^, and removal models using the package RMark ver. 2.2.4^45^.

#### Birds

To estimate the diversity and density of birds, we conducted point count surveys^46^. Each site had two point-count stations, spaced at least 200 m apart, that were surveyed on the same day. Counts were conducted at each site on three different days per season. To mitigate migration effects all counts were conducted between mid-June and mid-July. Bird surveys did not take place when it was raining, extremely cold (<0 degrees Celsius) or when wind speeds were over three on the Beaufort scale (>20 km\h). Wind speeds were obtained with handheld anemometers. Point counts began 15 minutes after sunrise and were completed by 10:00 AM, corresponding to when passerine birds are most active. Each survey consisted of one ten-minute count, split into two five-minute periods. Each individual bird was recorded only once over the entire count. Trained observers identified all birds to species by sight or sound and estimated the number of individuals of each species within 100 m of the sampling point. This gave us a sample area of 15,708 m^2^ at each site. Birds that flew through or were detected outside of the survey area or survey time were documented but not included in richness or density estimates. During 162 point-surveys we observed 1,039 birds from 52 species (107 individuals could not be identified).

The repeated point counts allowed us to estimate bird abundance and density using N-mixture models^47^. These models use repeated counts of individuals to estimate abundance within a sampling unit (in this case, the 100 m radius point count) while accounting for imperfect detection. Because abundance estimates refer to a set area, these can be converted to densities. Because data for some bird species were sparse, we fit data of all species jointly in a community model^48^. In community models, each species has its own set of parameters, but species-specific parameters are modeled as coming from a common underlying distribution that is shared by the community of species (essentially, a species-level random effect). This constitutes a form of information sharing, which improves parameter estimates for data-sparse species. In our community N-mixture model, we allowed for species-specific detection probabilities, as well as species-specific effects of burn category on abundance. We further included a fixed (across species) effect of the amount of wind during a given survey on detection, as wind can impair auditory detection of birds. We implemented the community N-mixture model in a Bayesian framework using the program JAGS ver. 4.2.0^49^ accessed through R with the package jagsUI ver. 1.5.0^50^. JAGS fits models using Markov chain Monte Carlo (MCMC) algorithms; we ran 100,000 MCMC iterations and discarded the first 50,000 as burn-in. We checked for chain convergence using the Gelman-Rubin statistic^51^; all parameters had a value <1.1, indicating convergence.

#### Large mammals

To estimate the abundance of mammals >2.5 kg we used camera traps. At each site, we deployed two Reconyx HC600 Hyperfire cameras, which have a no-glow infrared flash, preventing disturbance to wildlife and detection by humans. To reduce false triggers, we set cameras in shaded locations and cleared vegetation in front. Cameras were deployed along landscape features that suggested animal movement: game trails, forest openings, abandoned dirt roads, etc. Cameras were set at least 50 m apart and strapped to trees 40 cm above the ground and 1–2 m away from the edge of a trail or opening. Cameras were operated for 24 hours a day, with 3 consecutive pictures per trigger and no time lapse between triggers. All 54 camera traps were installed by early-July and retrieved in mid-September. Cameras were checked after four to six weeks for SD card and battery replacement. All pictures were reviewed manually for species identification; identified pictures of animals were organized into camera and species-specific folders for post-processing in the R computing environment ver. 3.4.3^52^ using the package camtrapR ver. 0.99.5^53^. Even though some bird and small mammal species were occasionally photographed by camera traps, we excluded their photo-records from further analysis. After adjusting for malfunction, cameras operated for 4,238 trapping nights over which they assembled 12,243 independent records (detections that were at least one hour apart if of the same species at the same camera) comprising 10 species of mammals >2.5 kg.

Because camera-trap images do not allow identification or counting of individuals for analysis with SCR or N-mixture models, we used the Royle-Nichols (RN) occupancy modeling framework^54^ to estimate abundance of medium/large mammal species. Occupancy models^55^ use repeated species detection/non-detection data from a collection of sampling locations to estimate species occurrence probability while accounting for imperfect detection. The RN model makes use of the fact that the probability of detecting a species at a site increases with the abundance of that species at that site, allowing estimation of site-level abundance from species level detection/non-detection data. We used the R package *camtrapR*^53^ to convert raw camera trap data for each species into a binary (detected = 1, not detected = 0) location-by-occasion format. Because of their proximity, we considered both cameras at a given plot to constitute a single sampling location. We defined an occasion as 10 consecutive days of sampling. To account for malfunctioning of some cameras, we calculated effort as the number of days per occasion that each pair of cameras was functional.

Because data for some species were sparse, we jointly analyzed data for all species in a community RN model (see Birds for a description of community models); we allowed for species-specific detection probabilities as well as species-specific effects of burn category on species abundance and included a fixed effect (across species) of effort on detection. The RN model returns sampling location level estimates of abundance, but in contrast to bird and reptile surveys, which were explicitly linked to a specific sampled area, the area sampled by a camera trap is not easily defined. Mammals recorded in the relatively small detection zone of a camera use much larger areas. We calculated densities of large mammals by dividing the abundance estimates from the RN model by the average home ranges of the recorded species. We fit the community RN model in a Bayesian framework as described for birds, running 30,000 MCMC iterations and discarding the first 15,000 as burn-in. All chains converged, according to the Gelman-Rubin statistic.

To obtain estimates for home range size and individual mass of large mammals, we searched the scientific literature for studies conducted in similar habitat (temperate coniferous forests) and for home ranges, during the summer and fall seasons. When no information from a similar habitat was available, we used studies from forested areas. When no information for target species was available, we used information from congeners. When multiple studies were available, we calculated the mean across studies, weighted by sample size if provided. Similarly, when studies provided information separately for males and females, we calculated a weighted mean. For some large mammal species, we only found information on annual home ranges, and to approximate a summer/fall home range, we multiplied these annual ranges by 0.67.

#### Bats

To survey bat community composition and abundance, we conducted acoustic sampling at each site. We placed a Wildlife Acoustics Songmeter SM4BAT FS echolocation detector at each site for a minimum of three consecutive nights. The detectors were set to record from sunset to sunrise when bats are most active. We attached microphones to t-posts placed in open areas, elevating them 2.5 meters from the ground to minimize signal attenuation from nearby vegetation and canopy. Bat calls were analyzed using SonoBat software to assess likely species for each file. These identifications were vetted by an experienced bat researcher, Ted Weller (USDA Forest Service). One site (B3U1) was not sampled with bat detectors, whereas two sites had one and two additional nights sampled, respectively. During 81 recorder nights, acoustic recorders captured 17,484 calls, of which 7348 were identified to 17 species.

Similar to camera traps, bat calls recorded by acoustic recorders do not allow counting or identifying individuals. Therefore, we built a community RN model to estimate abundance for bats. The model structure was identical to that described for medium/large mammals, except that it did not include any covariate on detection probability. Analogous to our medium/large mammal analysis, we used literature information on average home range size to convert bat abundance to bat density. We ran the bat model for 150,000 iterations and discarded the first 75,000 iterations as burn-in. All chains converged according to the Gelman-Rubin statistic.

#### Reptiles

To estimate community composition and density of reptiles, we conducted timed searches within the 90 m × 90 m small mammal trapping grids at each site. This gave us a sample area of 8100 m^2^ at each site. Reptile surveys were conducted either prior to small mammal trapping or two weeks post to minimize impacts from sampling disturbance. Reptile surveys were conducted between 8:00 am and 10:00 am by teams of two to four, for a total of one person-hour. We performed reptile surveys three times at each site, with at least one week between surveys. During searches, all refugia (i.e., rocks, logs) within the plot were carefully overturned and then replaced. In addition to reptile species, we documented time of day and weather, as well as cover type and cover length if applicable. Our reptile surveys yielded 145 records of two snake species and four lizard species. Two additional snake species were observed incidentally during other sampling methods.

Repeated counts of reptiles allowed us to use N-mixture models^47^ to estimate abundance and density. However, reptile data were extremely sparse and contained few species, precluding the use of a full community model as fit for birds. Instead, we structured reptile data into three groups: snakes (Western terrestrial garter snake (*Thamnophis elegans*) and Yellow-bellied racer (*Coluber constrictor*)), Alligator lizards (*Elgaria coerulea and E. multicarinata*) and Western fence lizards (*Sceloporus occidentalis*). We then combined data from all groups in a single N-mixture model, allowing for group-specific abundances. Due to sparse data, we only estimated an effect of burn category on abundance for fence lizards. Abundance for other species groups was assumed to be constant across burn categories in the model. To obtain species and burn category specific estimates of abundance, we combined model estimates of abundance with raw counts of individuals. For the two snakes, we calculated species level abundance by burn category by multiplying overall model-estimated snake abundance with the proportion of individuals made up by each species in each burn category. Because some *Elgaria* sp. observations could not be identified to species level, we did not attempt to calculate species specific abundances (but we note that the majority of observations was of *E. coerula*). We calculated species/group density by dividing abundance by the size of the sampling unit, in this case, a 90 × 90 m square. We fit the N-mixture model in R using the package unmarked ver. 0.12.2^56^.

### Species identification

Vertebrates were identified in the field or from photographs. Invertebrate specimens were collected and fixed in the field for identification in the lab where they were split into morphospecies using published keys^57,58^ and consultation with experts at the UC Davis Bohart Museum. Morphospecies were not always identified to lower taxonomic levels but were distinguished from similar species by coarse morphological characters. Prior to density estimation some morphospecies were aggregated based on taxonomy, body size, sample methodology, co-occurrence patterns and the difficulty in distinguishing members of the group. The rationales behind aggregations are documented in the raw invertebrate sampling data.

### Body size estimation

Food webs are commonly organized around body size^59–63^ and we include length and mass estimates for all life stages in our data set. We assume mean body size does not change substantially across burn severities and use a single estimate for all life stages regardless of treatment. When possible, we measured 10 individuals for each morphospecies and life stage (for invertebrates and small mammals only, as individuals from other taxonomic groups were not handled). Body mass for small mammals was measured directly with a Pesola scale. Body length estimates for invertebrates were derived from the longest measurement. Volumetric estimates were derived by applying the measured length, height and width to the three-dimensional shape that most closely approximates that of the organism (e.g. ellipsoid, rectangular prism, cylinder, hemisphere, cone). We estimated biomass by multiplying these volumetric estimates by a tissue density of 1.1 g/mL^30^.

Body sizes for organismal life stages observed but not captured (e.g. adult birds and large mammals) were estimated from the literature. Life stages for many organisms were not directly observed (e.g. lepidoptera eggs) but are almost certainly present. For these stages we again used the literature to estimate the body sizes. For example, we used a data set of egg sizes from 6,700 insect species to inform estimates for the species in our study^64^. The species list indicates the estimation method and literature source used to estimate the body size of each individual life stage is included in the species list.

### Links

Ecological networks can be broken into two types: Undirected and directed. Undirected networks include bipartite networks (e.g. plant-pollinator, host-parasite) and social-networks. In undirected networks, interactions (links) and their participants (nodes) are observed at the same time. Links are not inferred in undirected ecological networks (unless false-negatives due to sampling error are taken into account) because they are directly observed (e.g. tick removed from a lizard). Undirected links can be weighted (interaction strength) by counting observations.

Directed networks include food webs. Most food web links are inferred because it is not feasible to evaluate all possible consumer-resource interactions in a system through direct observation. The number of possible consumer-resource interactions in a food web is equivalent to the number of nodes squared. There were 3,084 nodes in the Eldorado National Forest, resulting in 9,511,056 potential consumer-resource interactions. In the scope of an ecological study, it is rarely feasible to directly observe most feeding links for most species^15^, much less an entire web, though molecular methods are bringing this closer to possibility for some guilds (e.g. large mammalian herbivores)^65^. While often detailed for birds and mammals, published accounts of direct observations of diets are often general (e.g. “Species A eats organic matter”) or entirely lacking for other groups. Restricting link assignments only to those that are directly observed will not create an accurate or unbiased food web.

To assign resources to consumers we supplemented our own field observations with published diet records, expert opinion and rule-based filters. In the absence of direct observation, rule-based filters are an important tool for sorting through the huge number of potential feeding interactions. Filters varied with the species and ontogenetic stages to which they were applied but consisted of two main types: Encounter and compatibility. Encounter filters determine the potential for consumers to interact with resources. Encounter filters are based on habitat co-occurrence, forging strategy and diel activity patterns. Applied to links that have passed through encounter filters, compatibility filters determine the outcome of a potential encounter. Compatibility filters are based on consumer diet information, consumer diet breadth, resource palatability, and consumer-resource body size ratios. For inclusion in the food web, a link must pass through both filters.

### Link assignment

All species were considered potential resources. For feasibility and reproducibility, species were broken into groups of encounter filters. First, we separated plants, fungi and detritus from other metazoans.

The first encounter filter was a loose group consisting of primary producers, non-living resources (e.g. detritus, carcasses), and saprophytes. In this first filter, primary producers were broken into parts (e.g. root, seed, leaves, etc). With few exceptions (i.e. moss, lichens, algae) primary producers in this filter group were evaluated as families or species. Saprophytes were evaluated as spores or adults. These groups served as the encounter filter for primary consumers and fungivores.

Next, we partitioned all other metazoans into smaller encounter filters based on phylogeny, behavior, activity, habitat and palatability. A species’ life stage can be a member of multiple resource groups. These groups served as the encounter filter for most predatory or omnivorous organism stages. These encounter filters included:

- Flying invertebrates
- Nocturnal flying invertebrates
- Diurnal flying invertebrates
- Non-flying invertebrates
- Ground-dwelling invertebrates
- Ground-dwelling invertebrates excluding spider eggs
- Soft-bodied ground dwelling invertebrates
- Invertebrates on plants
- Invertebrates on plants excluding spider eggs
- Soft-bodied invertebrates on plants
- Invertebrates on trees
- Reptiles
- Birds
- Small mammals
- Large mammals

Encounter filter exposure was tailored to consumer type. For consistency, consumers were broken into phylogenetic and ontogenetic guilds (e.g. lepidoptera larvae). For example, web-building spiders were exposed to flying insects, but not small-mammals encounter filters. Compatibility filters are specific to species and stage. For example, while both are exposed to the flying-insects encounter filter, adult and juvenile web-building spiders will have different compatibility filters because they have different consumer-resource body size ratios. Phylogenetic and ontogenetic consumer guilds are detailed below, but the decisions for each of the 178,655 links assignments in the food web can be found in and reproduced with the R code accompanying this manuscript

#### Spiders

As common, generalist insectivores, spiders are important consumers in the network^66^. To assign them resource links, spiders were broken into three ontogenetic stages: adult, egg, juvenile. Spiders were then separated into 17 consumer guilds by Family. The 87 spider species that could not be identified at the family level were assumed to be web-building spiders. To assign feeding interactions, we applied one or more invertebrate resource group filters (see above) to the members (species and stages) of each spider guild. Next, each link passed through a consumer-resource body size filter before being included in the network. For example, web-building spiders were assigned to capture flying insects and consume insects larger than 20% of their own body-length (as mesh size sets a lower limit of prey size) and less than 200% of their own body length^67^. Because of the generality of their filter-feeding hunting strategy, web-building spiders accounted for nearly half (32,663) of interactions with spiders as consumers. Additionally, when it was reported in the literature, adult members of a guild were assigned to consume nectar.

#### Herbivorous beetles

To assign consumer links to non-predatory beetles, their larval and adult stages were separated into Family-level feeding guilds. When information was available, links were evaluated at the species-stage level rather than the Family level. Resource links containing primary producers and non-living resources (detritus, carcasses, dung, etc) were assessed based on direct observation, published reports and expert opinions. When feeding on living plants, beetles were assigned to specific tissue types (e.g. flower, leaves, roots). For example, Cerambycidae_sp_2 was assigned to feed on the stems of *Arctostaphylos patula* as well as stems of plants from the family Rhamnaceae. This assigned 10,289 consumer links to herbivorous beetles.

#### Larval lepidoptera

Caterpillars were the most speciose herbivore group in the network. Larval lepidoptera vary greatly in host plant range and the quantity of information on their feeding habitats and were not lumped into guilds whenever possible. Microlepidoptera were not identified below the order level and were assumed to feed like Gelechiidae caterpillars (many species of which have been documented in Eldorado National Forest). We assigned links to caterpillars with resource filters of host plants and parts based on published diet records, palatability, expert opinion and observed co-occurrence between potential hosts and adults. This assigned 2,434 consumer links to caterpillars.

#### Adult lepidoptera

Adult lepidoptera feed primarily or exclusively on nectar from flowering plants. The host ranges for most adult lepidoptera are not well described. To assign nectar links, adult lepidoptera were grouped into families and passed through resource filters based on published records, co-occurrence, and expert opinion. Non-feeding adult moths were removed from link consideration. These filters assigned 5,854 nectar links to adult lepidoptera.

#### Adult hymenoptera as herbivores

Most species of hymenoptera are parasitoids as larvae and free-living as adults (eusocial hymenoptera are a notable exception). Many adult parasitic wasps supplement their protein intake with nectar and pollen. The plant host ranges for solitary adult wasps are not well known. We applied nectar and pollen resource filters to adult hymenoptera based on published records, co-occurrence and expert opinion. These filters assigned 4,880 links to adult hymenoptera.

#### Predatory beetles

Predatory and scavenging beetles are important consumers in terrestrial ecosystems. At the family level, the diets of predatory beetles are well-described relative to other arthropods. We grouped predatory and scavenging beetles into family guilds. We applied resource filters to predatory and scavenging beetles based on published records and expert opinion, habitat overlap, foraging strategy, palatability, and body size. These filters assigned 10,320 resource links to predatory beetles.

#### Flies

Flies in the Eldorado National Forest are a speciose group with diverse consumer strategies: herbivores, predators, scavengers, detritivores, parasitoids, and micropredators. Because of their trophic diversity each dipteran family was treated as its own guild, even then there were often large ontogenetic diet shifts across stages within a guild. We applied resource filters to dipterans based on published records and expert opinion of their diet. These filters were based on habitat overlap, palatability, and for predatory stages consumer-resource body size ratios. These filters assigned 9,541 resource links to flies.

#### Hymenopteran parasitoids

Parasitoid wasps are a diverse group that can have strong, even regulatory, effects on their host populations. We applied resource filters to hymenopteran parasitoid larvae in a three-step process. First, we used published records and expert opinion to establish their host range and host specificity. Next, we looked for the subset of potential hosts that co-occured with adult parasitoids across the most sites. Finally, we retained the subset of those host species whose adult body sizes were equivalent to or slightly larger than adult parasitoids. These filters assigned 4,695 host links to parasitoid wasps.

#### Other hymenoptera

The remaining hymenoptera were composed of wood wasps and gall wasps, as well as bees, predatory wasps and ants. Host plant links were assigned to wood wasps and gall wasps in the same way as larval lepidoptera. Nectar links were assigned to bees in the same way as adult lepidoptera. Prey links were assigned to solitary and eusocial wasps in the same way as insectivorous gleaning birds. Ants were treated as generalist omnivores whose links were assigned links in a manner similar to all of the above.

#### Hemiptera

Hemiptera were the most abundant consumer group in the Eldorado National Forest. Hemiptera use their proboscis to feed on fluids, either as herbivores or predators. To accommodate this variation in consumer strategies resource filters were assigned to individual hemiptera species based on phylogeny. Host plants were assigned to herbivores based on field observations, published records and expert opinion. Plant fluids (with the exception of sap from soft woods) were not included in the node list, so herbivores were assigned feeding links based on the plant tissues that they pierced (i.e. stem or leaves). Predatory hemiptera were assigned insects on plants passed through consumer-resource body size filters. When more specific dietary information was available resource filters were further refined. These filters assigned 3,177 links to the hemiptera

#### Collembola

From soil to canopy, springtails were widely distributed across Eldorado National Forest habitats and are important resources for arthropod secondary consumers. Collembola are herbivores, detritivores and fungivores. Little diet information is available for collembola, which were assigned resource filters based on habitat and phylogeny (order). Habitat was determined by collection method, and resource availability was determined by habitat. These, phylogenetic-habitat-resource filters assigned 140 links to the collembola.

#### Bark lice

Psocoptera were common on plants in the Eldorado National Forest, feeding on algae, lichen, moss, fungi and detritus. They exhibit little variation in diet and were assigned resource filters as a group at the Order level. This small set of resource filters assigned 120 links to the psocoptera.

#### Thrips

Thysanoptera were common and abundant consumers in the Eldorado National Forest, whose diets can range from herbivory to facultative predation and predation. To accommodate this variation in consumer strategy, resource filters were assigned to individual thysanoptera species based on phylogeny. Host plants for herbivores were based on field observations, supplemented with published records and expert opinion. Thysanoptera predators were assigned soft-bodied insects on plants passed through consumer-resource body size ratio filters. These resource filters assigned 924 links to thysanoptera.

#### Bats

Bats are important consumers of arthropods. Arthropod encounter filters were determined by bat foraging strategies reported in the literature. Juvenile bats were treated separately. Aerial hawking bats capture nocturnal flying insects. Gleaning bats capture insects on plants. Pallid bats (*Antrozous pallidus*) which fly close to the ground, capture arthropods on the ground and understory plants. These arthropod filters then passed through consumer-resource body size ratio filters, which assigned 4,621 links to adult bats.

#### Birds

Birds were the most speciose vertebrate group in the Eldorado National Forest. This diversity is reflected in their diets. Bird resource filters were based on published records and expert opinion. Herbivorous birds that were reported to feed on a type of plant tissue were assumed to feed on all tissues of that type, unless the literature indicated resource specialization. For example, if a bird was reported to feed on a fruit, it was assumed to feed on all fruit in the system. Resource links for predatory and omnivorous birds were passed through consumer-resource body size ratio filters. These filters assigned 56,584 links to birds as consumers.

#### Small mammals

Mammals <2.5 kg were common consumers in the Eldorado National Forest. Small mammal resource filters were based on published records and expert opinion. Herbivorous small mammals that were reported to feed on a type of plant tissue (e.g. seed) were assumed to feed on all plant tissues of that type, unless the literature indicated resource specialization. Resource links for omnivorous small mammals were passed through consumer-resource body size ratio filters. These filters assigned 4,648 links to small mammals as consumers.

#### Large mammals

Mammals >2.5 kg were comparatively uncommon but important consumers in the Eldorado National Forest. Large mammal resource filters were based on published records and expert opinion. Herbivorous large mammals that were reported to feed on a type of plant tissue were assumed to feed on all tissues of that type, unless the literature indicated resource specialization. Resource links for omnivorous and predatory large mammals were passed through consumer-resource body size ratio filters. These filters assigned 840 links to large mammals as consumers.

#### Juvenile mammals

Juvenile mammals were considered unweaned. Nursing mammals were included in the species list because they are more vulnerable than adults and are potential resources to different consumers as a result. Unweaned mammals “feed” on their mothers, who are considered their only resource link. Nursing filters assigned 38 links to juvenile mammals.

#### Reptiles

While not diverse or common, reptiles on the mountain slopes of Eldorado National Forest are potentially important consumers for many groups. Reptile resource filters were assigned based on published records and expert opinion. All resource links were passed through consumer-resource body size ratio filters. These filters assigned 1,487 links to reptiles.

#### Mites

Mites are ubiquitous and important consumers in many habitats but are often overlooked because of their small size and taxonomic difficulty. Mites were treated as consumer groups based on taxonomy. Resources for predatory mites were based on habitat and passed through consumer-resource body size ratios filters. Resource filters assigned 2,971 links to mites.

#### Miscellaneous arthropods

A few arthropods not discussed above were not speciose enough to be dealt with separately here. These remaining miscellaneous arthropods were treated as groups at various taxonomic levels. Encounter and compatability filters for these groups were based on published records. Encounter filters for predatory (centipedes, odonates, pseudoscorpions, solfugids, mantids, opiliones, neuroptera, embioptera, pauropoda, eusocial wasps) and omnivorous (dermaptera) groups were based on habitat and passed through consumer-resource body size filters. Encounter filters were assigned by habitat for detritivores, herbivores and fungivores (archeognatha and isopods). These filters assigned 3,157 links to miscellaneous arthropods as consumers.

## Data Records

All our data and code^68^ are data available in the Dryad Digital Repository: 10.5061/dryad.rv15dv47g.

The data are organized into three compressed directories:

### Acoustic_surveys

This directory is 162.95 GB and contains WAV files from bat acoustic surveys. WAV files are organized into subdirectories by location (site name), collection date (DAY_MONTH_YEAR), and receiver ID number. Subdirectories >10 GB were broken into 2 or more parts (e.g. A, B, C.). WAV file names contain metadata: Receiver ID, collection date (YEARMONTHDAY) and collection time (HRMINSEC). Detailed methods on data collection and processing are provided above.

### Assembly_workflow

This directory is 14.97 MB and contains 134 items: R files, CSV data files, and an RTF supplemental bibliography. Online-only Table 1 describes the format and content of each file. The R files rely on the CSV and Rdata files to (1) estimate biomass density, (2) apply resource filters to identify consumer links and (3) assemble food webs. There are four types of data files: Raw, Temporary, Tidy and Summary. Raw data is as close as possible to the form in which it was collected. As such, raw data is not ready for analysis. We have provided tidy versions of each raw data set that are ready for analysis. Temporary data files are included for transparency but are used to create summary data files, and not intended to be informative as standalone products. Summary data files provide estimated biomass densities and network structure for each of our three burn categories. All tidy and summary data files are accompanied by metadata files describing their contents. The code to transform raw data files into tidy, temporary, and summary files is included in the R files. CSV and Rdata files are organized by sampling method and target group of organisms as described above.

**Table 1:** Sample coverage estimates including 95% confidence intervals, individuals sampled and species richness for all species and taxonomic groups in each of three burn severity categories in the Eldorado National Forest, California, three years after the King Fire.

Taxonomic Group	Treatment	Individuals	Observed Richness	Sample Coverage	Lower CL	Upper CL
All Species	Unburned	31216	612	0.995	0.995	0.996
All Species	Moderate	12708	509	0.989	0.987	0.990
All Species	Severe	16505	429	0.992	0.991	0.993
Bats	Unburned	5077	16	1.000	0.999	1.000
Bats	Moderate	2054	15	1.000	0.999	1.000
Bats	Severe	4305	14	1.000	1.000	1.000
Birds	Unburned	658	41	0.991	0.986	0.996
Birds	Moderate	728	44	0.990	0.985	0.996
Birds	Severe	550	36	0.998	0.993	1.004
Invertebrates	Unburned	19856	485	0.993	0.992	0.994
Invertebrates	Moderate	5096	389	0.974	0.970	0.977
Invertebrates	Severe	3497	325	0.964	0.960	0.968
Large mammals	Unburned	4248	8	1.000	1.000	1.000
Large mammals	Moderate	3378	8	1.000	1.000	1.000
Large mammals	Severe	4617	6	1.000	1.000	1.000
Plants	Unburned	1077	48	0.998	0.995	1.001
Plants	Moderate	1172	42	0.997	0.995	1.000
Plants	Severe	2982	38	0.999	0.998	1.000
Reptiles	Unburned	15	5	0.876	0.740	1.011
Reptiles	Moderate	15	3	1.000	0.954	1.046
Reptiles	Severe	116	3	1.000	0.996	1.004
Small mammals	Unburned	285	9	0.997	0.990	1.003
Small mammals	Moderate	265	8	1.000	0.996	1.004
Small mammals	Severe	438	7	0.998	0.993	1.002

Groups were determined by sampling methods. Methods that had overlapping species records were grouped together.

### Camera_trap_data

This directory is 5.59 GB and contains JPG files from camera traps. JPG files are organized into subdirectories by type of animal captured (e.g. bird, coyote, squirrel, etc.). These then aggregated by location (site name) and camera trap ID (i.e. CT1 or CT2). JPG file names contain metadata: Site name, camera trap ID, collection date (DAYMONTHYEAR) and image number. Detailed methods on data collection and processing are described above.

## Technical Validation

### Species Inclusion

**Fig. 4:**
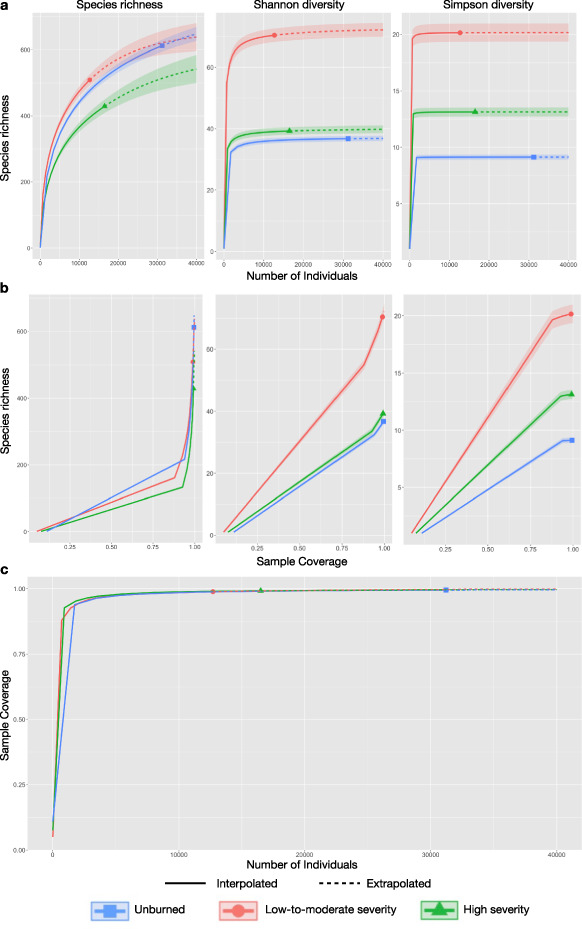
Rarefaction, extrapolation and coverage estimates for all species. Estimates include all 60,429 individual organisms (producers and consumers) encountered during sampling of the Eldorado National Forest, California, three years after the King Fire. Color indicates burn severity: blue = unburned, red = low-to-moderate severity, green = high severity. Line type indicates estimate: interpolation (solid), extrapolation (dashed). (**a**) Sample-size based rarefaction and extrapolation. Burn severity impacts richness and evenness. Burned areas have fewer individual organisms but more common (Shannon diversity) and dominant species (Simpson diversity). (**b**) Coverage-based rarefaction and extrapolation. High coverage of all species, common species (Shannon diversity), and dominant species (Simpson diversity) suggests larger sample sizes may add rare species, but relative differences between treatments are unlikely to change. (**c**) Sample coverage estimates. Sample completeness estimates are > = 99% across burn severities, indicating comprehensive and comparable sampling effort.

To estimate the coverage of our sampling methods we conducted interpolation and extrapolation analysis of Hill numbers (effective number of species) using the “iNEXT” package in R^69–71^. Hill numbers^72^ integrate richness and abundance data (calculated across a gradient, *q*, of how much weight is given to rare species), into a series of diversity measures including species richness, Shannon diversity^73^ and Simpson diversity^69^ and are a consensus best-practices diversity measure^74^. Shannon diversity and Simpson diversity can be interpreted as estimates of the number of common species and the number of dominant species, respectively. To estimate diversity and evaluate coverage, we grouped sampling methods with overlapping species records at the site level (Table 1, Online-only Table 2). For example, shrub transects, tree transects, and box quadrat data were grouped to estimate primary producer richness. To estimate total coverage and richness we also combined all species (producers and consumers) across all methods at each site (Fig. 4).

Burn severity impacts species richness and evenness. Burned sites have fewer individual organisms but more species are common or dominant (Fig. 4a, Online-only Table 2). At larger sample sizes low-to-moderate severity sites may be comparable to unburned sites in species richness (Fig. 4a), but coverage-based analyses (Fig. 4b,c) suggest this is unlikely.

Sample coverage estimates (Fig. 4c,Table 1), and coverage-based rarefaction and extrapolation curves (Fig. 4b) suggest the observed differences in richness across treatments are not sampling artifacts. Across burn-severities, sample coverage estimates for all species combined were high, >=99% (Fig. 4c, Table 1). All three burn-severity treatments were within 1% of each other’s estimated completeness (Table 1), which makes standardization for comparison straightforward^73^. Within taxonomic groups, sample coverage estimates were also high, all >96% (Table 1) with narrow confidence intervals. Sample coverage was consistent within groups, treatments were within 3% of each other (Table 1). The species-poor reptiles in unburned landscapes are an exception and the coverage estimate (88%) suggests they were under-sampled. The impact of this on the system description is small as reptiles comprised <1% of species in unburned areas and did not make significant contributions to biomass.

High coverage of all species, common species, and dominant species (Fig. 4b) suggests that larger sample sizes may add rare species, but the relative differences between burn treatments are unlikely to change. Within taxonomic groups, extrapolated diversity estimates also suggest that differences across burn severities are maintained at larger sample sizes (Online-only Table 2). Notably, our observed species counts were close to counts reported elsewhere. For example, other richness surveys in the Sierra Nevada montane forests reported 51 birds^75^ compared to our 52 bird species. Further, species that were not captured in our sampling are likely low-density and not substantial contributors to ecosystem energetics.

### Species Quantification

We include measures of variability, in addition to the mean estimates presented in our summarized food web data. When two measures gave density estimates for a particular species, we chose the measure with the greatest ability to detect the species. Standard error and sample size are included for body size estimates. References are included for all body size estimates obtained from the literature. The filters used to assign each of the 178,655 links are included in the relevant R scripts.

### Link Inclusion

We included 178,655 or 19% of the >9.5 million potential links in the network. This is within the range of connectance reported for other well studied^76^ and speciose food webs^77–81^.

We have attempted to set the new standard for transparency in our link assignment methods^16^ by providing the following:

- Supplementary bibliography of the 425 primary literature sources and databases used to augment our direct observations and inform link assignments.
- R code containing the rationale used to assign each of the 178,655 links in the network
- R code to assemble the food webs for each burn category from scratch.

To our knowledge, this exceeds the standard of even the most well-documented published food webs and is the most-detailed description of the methods for assigning links yet published^78,82–84^. The R code was designed to be a tool for researchers and managers. Researchers can adjust any filter (searchable by consumer species and family) and rerun the assembly code with these changes to alter network structure. For example, one of our compatibility filters assumes the body length of the salticid spider prey is 25–75% of the spider’s body length^85^. If a researcher wanted to narrow/widen this range they can simply change this parameter and rerun the code. This will allow this data to remain easily useable even if new findings change our understanding of the best rules used to assign feeding links.

### Link quantification

Links in this data set are binary, denoting only presence or absence. This means that diet preferences are ignored and all feeding links are regarded as equally important, which is certainly not the case. There are a variety of ways to add interaction strength estimates to link assignments^86,87^. However, such estimates are theoretical and beyond the scope of this data set.

## Usage Notes

### Species aggregation

A limitation of the data set is aggregation of the species comprising some primary producers and saprophytes into single representative nodes. This occurred when members of groups where known to be present but not explicitly surveyed. These aggregated nodes do not include biomass information and likely conceal considerable diversity. For example, 1575 lichens^88^ and 596 mosses^89^ are reported from California, but are represented by a single node each in our data set. Bacteria, saprophytic fungi, mycorrhizae, and nematodes are understudied groups often neglected in food web studies, including this one. These groups are important consumers (and symbionts) and their likely contributions to diversity and biomass are not reflected in the data set. For example, soil nematode abundance peaks globally in temperate coniferous forests^90^.

### Species omission

A second limitation of the data set is the absence of certain groups. Our survey methods captured many nocturnal animals (black light, small mammal traps, acoustic bat surveys, camera traps) but did not collect any information on nocturnal birds such as owls. Eldorado National Forest is within the year-round or breeding range of several owl species. Small mammal traps were primarily open at night, making estimates of diurnal mammals less accurate. We have included insects as parasites on plants^91^ (i.e. lepidoptera larvae) and other insects (i.e. parasitoid wasps) but have omitted all other parasites (e.g. helminths) and endophytes. Parasites are important contributors to ecosystem structure^77,92^ and dynamics^31,93^ and future augmentations to include them would improve the accuracy of the web.

### Temporal limitation

The data set is temporally limited at both large and small scales. The data set represents a snapshot in time of the species present during the summer months three years post-fire. Three years was selected as a time long enough since the initial fire to provide time for species recolonization and some system recovery. However, three-years is also close enough to the burn to be able to compare the effects of burn severity on recovery which can be hard to distinguish in the longer term (i.e. 15 years) post-fire^94–96^. The Sierra Nevada in California is a highly seasonal system, but summer sampling probably captures peak abundance, diversity and near-peak productivity^97^. The limited seasonality of sampling certainly contributes to species omissions detailed above - as we will have missed many migratory species and seasonally dormant species. As treatments were sampled simultaneously, this should have relatively small impact on comparisons across treatments and should accurately represent interactions occurring in the summer.

### Large mammal and bat densities

Using a RN model to estimate abundance, then converting that into density by dividing it by the average home range, is an approach that relies on a multitude of assumptions (about the abundance-detection relationship, individual detectability, movement and distribution, as well as parametric assumptions) that cannot be tested with the data we have at hand. Similarly, by using information on home range size from different study areas and in some cases, on closely related but different species, we make further assumptions about how representative that information is for the species in our study system. We do not recommend this as an approach for density estimation in general, but in this system-wide study, we did not have the means to collect adequate data for proper abundance estimation (repeated counts, capture-recapture data) for all species of interest. Any bias introduced into biomass estimates due to this approach for density estimation should be comparable across the three burn categories, which constitute our main dimension of interest for ecological comparison. We are therefore confident that even though our density estimates for bats and medium/large mammals may be biased to an unknown degree, comparisons related to these estimates of biomass across burn categories remain valid.

### Taxonomic resolution

Many groups would benefit from increased taxonomic resolution of their members. For example, there are 87 spider morphospecies that we were not able to determine below the order level. Without any other information the 87 unknown spiders were assumed to be web-building, but that is unlikely to be the case for all of them. Improving the taxonomic resolution of these and other nodes would increase the precision with which we can assign them resource filters.

### Link resolution

Resolution of trophic interactions could be improved by future sampling efforts. Advances in molecular methods are making it possible to distinguish the diets of entire guilds^65^. Applying this approach to vertebrates in the system (even if just for plant tissues in their diets) would improve trophic resolution for this group. Refining resource filters with field observations is another way to improve trophic resolution. Developing a database of species affiliations for insects on plants would allow us to refine and partition the very broad “Arthropods on plants” filters. For example, if certain hunting spiders are only affiliated with certain plants, we could restrict their resource filters to include only insects found on those plants rather than insects on all plants. Consumer-resource trials in mesocosms are another way to improve dietary information for predatory consumers.

## Data Availability

We have included the code necessary to reproduce all our work^68^. We have broken the code into three scripts: • 01_Biomass_density_estimation.R: Converts raw data files into tidy formats and generate summary estimates of species biomasses. Biomass density estimates were conducted in R^52^. • 02_Link_Assignment.R: Generates encounter and compatibility filters and assigns feeding links to all consumers. Resource filters were assigned with the *tidyverse* package^57^. • 03_Web_Assembly.R: Assembles food webs for each burn category into analyzable network format. Network assembly was conducted with the *igraph* package^98^. R scripts are in the Assembly_workflow folder available in the Dryad Digital Repository^68^: 10.5061/dryad.rv15dv47g.

## Online-only Tables

**Online-only Table 1:** Assembly workflow file description.

File_name	File_format	Description
01_Biomass_density_estimation	R	Estimates abundances and biomass densities from raw data files. This also creates most of the Temporary, Tidy and Summary files.
02_Link_Assignment	R	Generates a list of feeding links by assigning resource filters to each consumer node. This creates the summary links lists.
03_Web_Assembly	R	Assemble and visualize food webs created with the above code.
Cheddar_webs	csv	A folder containing webs formatted to estimate prey averaged trophic level for each node in burn severity.
Raw_Bat_Abundance_Biomass	csv	Acoustic observations and abundance estimates of bats at each sampling site.
Raw_Bat_Abundance_Biomass2	csv	Acoustic observations and abundance estimates of bats at each sampling site.
Raw_Bat_Data	csv	Metadata for bat acoustic records.
Raw_Bird_Abundance_Biomass	csv	Observations and abundance estimates of birds at each sampling site.
Raw_Bird_Abundance_Biomass2	csv	Observations and abundance estimates of birds at each sampling site.
Raw_Bird_Data	csv	Bird observations containing metadata.
Raw_Box_Quadrat	csv	Observations and volume estimates of plant matter from box quadrats at each site.
Raw_Camera_Data	csv	Metadata for camera trap records from each site. Observations are identfied by organismal group.
Raw_Camera_Trap_Functioning	csv	Metadata on the camera traps themselves.
Raw_Canopy_Cover_Data	csv	Canopy cover estimates from tree transects.
Raw_DBH_Height_Parameters	csv	Parameters used to estimate height from DBH for tree species.
Raw_Fogging_Metadata	csv	Metadata describing fogs.
Raw_Hardwood_Tissue_Parameters	csv	Parameters used to estimate tissue masses for hardwood trees.
Raw_Invertebrate_Body_Size	csv	Morphometric measurements of invertebrates.
Raw_Invertebrate_Data	csv	Counts and mass estimates for all invertebrates observed.
Raw_Large_Mammal_Abundance_Biomass	csv	Observations of large mammals from camera traps, including abundance and biomass density estimates.
Raw_Large_Mammal_Abundance_Biomass2	csv	Observations of large mammals from camera traps, including abundance and biomass density estimates.
Raw_Reptile_Abundance_Biomass	csv	Observations of reptiles from transects, including abundance and biomass density estimates.
Raw_Reptile_Abundance_Biomass2	csv	Observations of reptiles from transects, including abundance and biomass density estimates.
Raw_Reptile_Data	csv	Reptile observations containing metadata.
Raw_Sample_Plot_Locations	csv	Metadata for sampling sites.
Raw_Shrub_Biomass	csv	Shrub volume and mass estimates from transects.
Raw_Sites_Metadata	csv	Key to site names used in raw files and physical datasheets.
Raw_Small_Mammal_Abundance_Biomass.csv	csv	Observations of small mammals from Sherman traps, including abundance and biomass density estimates.
Raw_Small_Mammal_Abundance_Biomass2	csv	Observations of small mammals from Sherman traps, including abundance and biomass density estimates.
Raw_Small_Mammal_Data	csv	Small mammal observations containing metadata.
Raw_Softwood_Tissue_Parameters	csv	Parameters used to estimate tissue masses for softwood trees.
Raw_Species_Key	csv	Key to species names used in raw files and physical datasheets.
Raw_Transect_Ground_Cover	csv	Height and cover measurements for understory vegetation and detritus.
Raw_Transect_Shrub	csv	Volumetric measurements for shrubs on transects.
Raw_Transect_Tree	csv	DBH and qualitative descriptors of trees on transects.
Raw_Tree_Biomass	csv	DBH and qualitative descriptors of trees on transects, including tissue mass estimates.
Raw_Tree_DBH_Biomass_Parameters	csv	Parameters used to estimate biomass from DBH for tree species.
Raw_Tree_Transect_Data	csv	DBH and qualitative descriptors of trees on transects.
Raw_Woody_Debris_Biomass_Density	csv	Wooody debris biomass density estimates at each site.
Raw_Woody_Debris_Data	csv	Wooody debris observations, measurements and metadata.
Raw_Woody_Debris_Decay_Correction	csv	Density correction for state of decay in woody debris.
Summary_Abundance_Biomass_Data	csv	Abundance and biomass density estimates for each species stage observed in each treatment.
Summary_Abundance_Biomass_Metadata	csv	Column descriptors for Summary_Abundance_Biomass_Data.
Summary_All_Links_Metadata	csv	Column descriptors for Summary_All_Links.
Summary_All_Links	csv	All possible consumer-resource interactions, identified across all treatments.
Summary_All_Nodes_List_Metadata	csv	Column descriptors for Summary_All_Nodes_List.
Summary_All_Nodes_List	csv	All species and lifestages observed and inferred across all treatments.
Summary_High_Severity_Links_Metadata	csv	Column descriptors for Summary_High_Severity_Links.
Summary_High_Severity_Links	csv	All possible consumer-resource interactions, identified in sampling sites that experienced high severity burns.
Summary_High_Severity_Nodes_Metadata	csv	Column descriptors for Summary_High_Severity_Nodes.
Summary_High_Severity_Nodes	csv	All species and lifestages observed and inferred in sampling sites that experienced high severity burns.
Summary_Moderate_Severity_Links_Metadata	csv	Column descriptors for Summary_Moderate_Severity_Links.
Summary_Moderate_Severity_Links	csv	All possible consumer-resource interactions, identified in sampling sites that experienced moderate severity burns.
Summary_Moderate_Severity_Nodes_Metadata	csv	Column descriptors for Summary_Moderate_Severity_Nodes.
Summary_Moderate_Severity_Nodes	csv	All species and lifestages observed and inferred in sampling sites that experienced moderate severity burns.
Summary_Node_Taxonomy_Metadata	csv	Column descriptors for Summary_Node_Taxonomy.
Summary_Node_Taxonomy	csv	Morphospecies taxonomy.
Summary_Unburned_Links_Metdata	csv	Column descriptors for Summary_Unburned_Links.
Summary_Unburned_Links	csv	All possible consumer-resource interactions, identified in sampling sites that were not burned.
Summary_Unburned_Nodes_Metadata	csv	Column descriptors for Summary_Unburned_Nodes.
Summary_Unburned_Nodes	csv	All species and lifestages observed and inferred in sampling sites that were not burned.
Supplemental_bibliography_link_assignments	rtf	A 425 item biobliography of source used to inform link assignments.
Temporary_Box_Quadrat_Data	Rdata	Observations and volume estimates of plant matter from box quadrats at each site formatted for tidying.
Temporary_Ground_Cover_Data	Rdata	Height and cover measurements for understory vegetation and detritus formatted for tidying.
Temporary_Invertebrate_Biomass	csv	Invertebrate abundance and biomass estimates formatted for tidying.
Temporary_Invertebrate_Sampling_Data	csv	Invertebrate observations formatted for tidying.
Temporary_Invertebrate_Species_List	csv	Invertebrate species list formatted for tidying.
Temporary_Invertebrate_Taxonomy_List	Rdata	Taxonomy of invertebrate morphospecies formatted for tidying.
Temporary_Invertebrate_Taxonomy_Metadata	csv	Column descriptors for Temporary_Invertebrate_Taxonomy_List formatted for tidying.
Temporary_Invertebrate_Taxonomy	csv	Taxonomy of invertebrate morphospecies formatted for tidying.
Temporary_Invertebrate_Taxonomy	Rdata	Taxonomy of invertebrate morphospecies formatted for tidying.
Temporary_Plant_Biomass	csv	Plant biomass estimates formatted for tidying.
Temporary_Plant_Species_List	Rdata	List of plant species formatted for tidying.
Temporary_Plant_Taxonomy_Metadata	csv	Column descriptors for Temporary_Plant_Taxonomy formatted for tidying.
Temporary_Plant_Taxonomy	csv	Taxonomy of plant morphospecies formatted for tidying.
Temporary_Shrub_Data	Rdata	Shrub volume and mass estimates from transects formatted for tidying.
Temporary_Sites_Metadata	Rdata	Key to site names used in raw files and physical datasheets formatted for tidying.
Temporary_Taxonomy_List	Rdata	Morphospecies taxonomy formatted for tidying.
Temporary_Tree_Data	Rdata	Tree observations and biomass estimates formatted for tidying.
Temporary_Vertebrate_Biomass_Data	csv	Vertebrate biomass estimates formatted for tidying.
Temporary_Vertebrate_Taxonomy	csv	Vertebrate morphospecies taxonomy formatted for tidying.
Temporary_Vertebrate_Taxonomy	Rdata	Vertebrate morphospecies taxonomy formatted for tidying.
Tidy_Bat_Data	csv	Acoustic bat observations formatted for analysis.
Tidy_Bat_Metadata	csv	Column descriptors for Tidy_Bat_Metadata formatted for analysis.
Tidy_Bird_Count_Data	csv	Bird observations formatted for analysis.
Tidy_Bird_Count_Metadata	csv	Column descriptors for Tidy_Bird_Count_Data formatted for analysis.
Tidy_Box_Quadrat_Data	csv	Observations and volume estimates of plant matter from box quadrats at each site formatted for analysis.
Tidy_Box_Quadrat_Metadata	csv	Column descriptors for Tidy_Box_Quadrat_Data formatted for analysis.
Tidy_Camera_Trap_Data	csv	Camera trap records from each site. Observations are identfied by species.
Tidy_Camera_Trap_Function_Data	csv	Metadata on the functioning of the camera traps themselves.
Tidy_Camera_Trap_Function_Metadata	csv	Column descriptors for Tidy_Camera_Trap_Function_Data formatted for analysis.
Tidy_Camera_Trap_Metadata	csv	Column descriptors for Tidy_Canopy_Cover_Dataformatted for analysis.
Tidy_Canopy_Cover_Data	csv	Canopy cover estimates from tree transects formatted for analysis.
Tidy_Canopy_Cover_Metadata	csv	Column descriptors for Tidy_Canopy_Cover_Data formatted for analysis.
Tidy_Coarse_Woody_Debris_Data	csv	Wooody debris observations, measurements and metadata formatted for analysis.
Tidy_Coarse_Woody_Debris_Metadata	csv	Column descriptors for Tidy_Coarse_Woody_Debris_Data formatted for analysis.
Tidy_Fogging_Data	csv	Metadata describing fogs formatted for analysis.
Tidy_Fogging_Metadata	csv	Column descriptors for Tidy_Fogging_Data formatted for analysis.
Tidy_Ground_Cover_Data	csv	Height and cover measurements and biomass estimates for understory vegetation and detritus formatted for analysis.
Tidy_Ground_Cover_Metadata	csv	Column descriptors for Tidy_Ground_Cover_Data formatted for analysis.
Tidy_Invertebrate_Sampling_Data	csv	Invertebrate observations, counts and body size estimates formatted for analysis.
Tidy_Invertebrate_Sampling_Metadata	csv	Column descriptors for Tidy_Invertebrate_Sampling_Data formatted for analysis.
Tidy_Invertebrate_Size_Data	csv	Morphometric measurements of invertebrates formatted for analysis.
Tidy_Invertebrate_Size_Metadata	csv	Column descriptors for Tidy_Invertebrate_Size_Data formatted for analysis.
Tidy_Invertebrate_Taxonomy_Metadata	csv	Column descriptors for Tidy_Invertebrate_Taxonomy formatted for analysis.
Tidy_Invertebrate_Taxonomy	csv	Taxonomy of invertebrate morphospecies formatted formatted for analysis.
Tidy_Reptile_Data	csv	Reptile observations and counts, containing metadata formatted for analysis.
Tidy_Reptile_Metadata	csv	Column descriptors for Tidy_Reptile_Data formatted for analysis.
Tidy_Sample_Plot_Location_Data	csv	Sampling site metadata formatted for analysis.
Tidy_Sample_Plot_Location_Metadata	csv	Column descriptors for Tidy_Sample_Plot_Location_Data formatted for analysis.
Tidy_Shrub_Data	csv	Volumetric measurements and biomass estimates for shrubs on transects formatted for analysis.
Tidy_Shrub_Metadata	csv	Column descriptors for Tidy_Shrub_Data formatted for analysis.
Tidy_Sites_Data	csv	Key to revised site names formatted for analysis.
Tidy_Sites_Metadata	csv	Column descriptors for Tidy_Sites_Data formatted for analysis.
Tidy_Small_Mammal_Data	csv	Small mammal observations and measurements containing metadata formatted for analysis.
Tidy_Small_Mammal_Metadata	csv	Column descriptors for Tidy_Small_Mammal_Data formatted for analysis.
Tidy_Tree_Data	csv	Tree observations and biomass estimates formatted for analysis.
Tidy_Tree_Metadata	csv	Column descriptors for Tidy_Tree_Data formatted for analysis.
Tidy_Vegetation_Box_Data	csv	Observations and volume estimates of plant matter from box quadrats at each site formatted for analysis.
Tidy_Vegetation_Box_Metadata	csv	Column descriptors for Tidy_Vegetation_Box_Data formatted for analysis.
Tidy_Vegetation_Cover_Data	csv	Volumetric measurements and biomass estimates for understory vegetation and detritus formatted for analysis.
Tidy_Vegetation_Cover_Metadata	csv	Column descriptors for Tidy_Vegetation_Cover_Data formatted for analysis.

Estimates of species richness, Shannon diversity and Simpson diversity, including standard error and 95% confidence intervals for all species and taxonomic groups in each of three burn severity categories in the Eldorado National Forest, California, three years after the King Fire. Groups were determined by sampling method. Methods that had overlapping species records were grouped together. Within taxonomic groups, extrapolated diversity estimates suggest that differences across burn severities are maintained at larger sample sizes.

**Online-only Table 2:** Sample-size-based extrapolation.

Taxonomic Group	Treatment	Individuals	Observed Richness	Sample Coverage	Lower CL	Upper CL
All Species	Unburned	31216	612	0.995	0.995	0.996
All Species	Moderate	12708	509	0.989	0.987	0.990
All Species	Severe	16505	429	0.992	0.991	0.993
Bats	Unburned	5077	16	1.000	0.999	1.000
Bats	Moderate	2054	15	1.000	0.999	1.000
Bats	Severe	4305	14	1.000	1.000	1.000
Birds	Unburned	658	41	0.991	0.986	0.996
Birds	Moderate	728	44	0.990	0.985	0.996
Birds	Severe	550	36	0.998	0.993	1.004
Invertebrates	Unburned	19856	485	0.993	0.992	0.994
Invertebrates	Moderate	5096	389	0.974	0.970	0.977
Invertebrates	Severe	3497	325	0.964	0.960	0.968
Large mammals	Unburned	4248	8	1.000	1.000	1.000
Large mammals	Moderate	3378	8	1.000	1.000	1.000
Large mammals	Severe	4617	6	1.000	1.000	1.000
Plants	Unburned	1077	48	0.998	0.995	1.001
Plants	Moderate	1172	42	0.997	0.995	1.000
Plants	Severe	2982	38	0.999	0.998	1.000
Reptiles	Unburned	15	5	0.876	0.740	1.011
Reptiles	Moderate	15	3	1.000	0.954	1.046
Reptiles	Severe	116	3	1.000	0.996	1.004
Small mammals	Unburned	285	9	0.997	0.990	1.003
Small mammals	Moderate	265	8	1.000	0.996	1.004
Small mammals	Severe	438	7	0.998	0.993	1.002

The name, type and contents description for each of the 135 files in the Assembly_workflow folder. This folder contains all the raw, tidy, summary and meta data and code needed to reproduce the data set. This includes density and body size estimates for species in the Eldorado National Forest, California, three years after the King Fire. It also includes the files to assemble food webs for each of three burb categories: unburned, low-to-moderate severity, severe.
